# Supplementary material for: A versatile toolbox for determining IRES activity in cells and embryonic tissues
Source: EMBO J. 2025 Mar 13;44(9):2695–724. doi: 10.1038/s44318-025-00404-5 (PMC12048685; doi:10.1038/s44318-025-00404-5)
Supplement: Supplementary file 1 — Appendix [file 44318_2025_404_MOESM1_ESM.pdf]

Appendix for

A versatile toolbox for determining IRES activity in cells and embryonic tissues

Philipp Koch[1*], Zijian Zhang[2*], Naomi R. Genuth[2,3*], Teodorus Theo Susanto[2,4], Martin Haimann[1], Alena Khmelinskaia[5,6,7], Gun Woo Byeon[2,8], Saurabh Dey[1], Maria Barna[2‡] and Kathrin Leppek[1‡]

[1] Institute of Clinical Chemistry and Clinical Pharmacology, Biomedical Center II (BMZ II), Venusberg-Campus 1, University Hospital Bonn, University of Bonn, 53127 Bonn, Germany
[2] Department of Genetics, Stanford University, Stanford, California 94305, USA
[3] Present address: Department of Molecular and Cell Biology, Howard Hughes Medical Institute, University of California, Berkeley, Berkeley, CA 94720, USA
[4] Present address: Epigenetic and Epitranscriptomic Systems, Genome Institute of Singapore, A*STAR, Singapore 138672, Singapore
[5] Transdisciplinary Research Area "Building Blocks of Matter and Fundamental Interactions", University of Bonn, 53113 Bonn, Germany
[6] Life and Medical Sciences Institute, University of Bonn, 53121 Bonn, Germany
[7] Present address: Department of Chemistry, Ludwig-Maximilians-Universität München, 81377 München, Germany
[8] Present address: Department of Electrical and Computer Engineering, University of Washington, Seattle, WA, USA 98195, USA

Contact: Maria Barna: mbarna@stanford.edu; Kathrin Leppek: kleppek@uni-bonn.de
* authors contributed equally
‡ co-corresponding authors

Table of Contents

This document includes: page

Appendix Figure S1 to S6 2-13
Appendix Table S1 14
Appendix Table S2 16

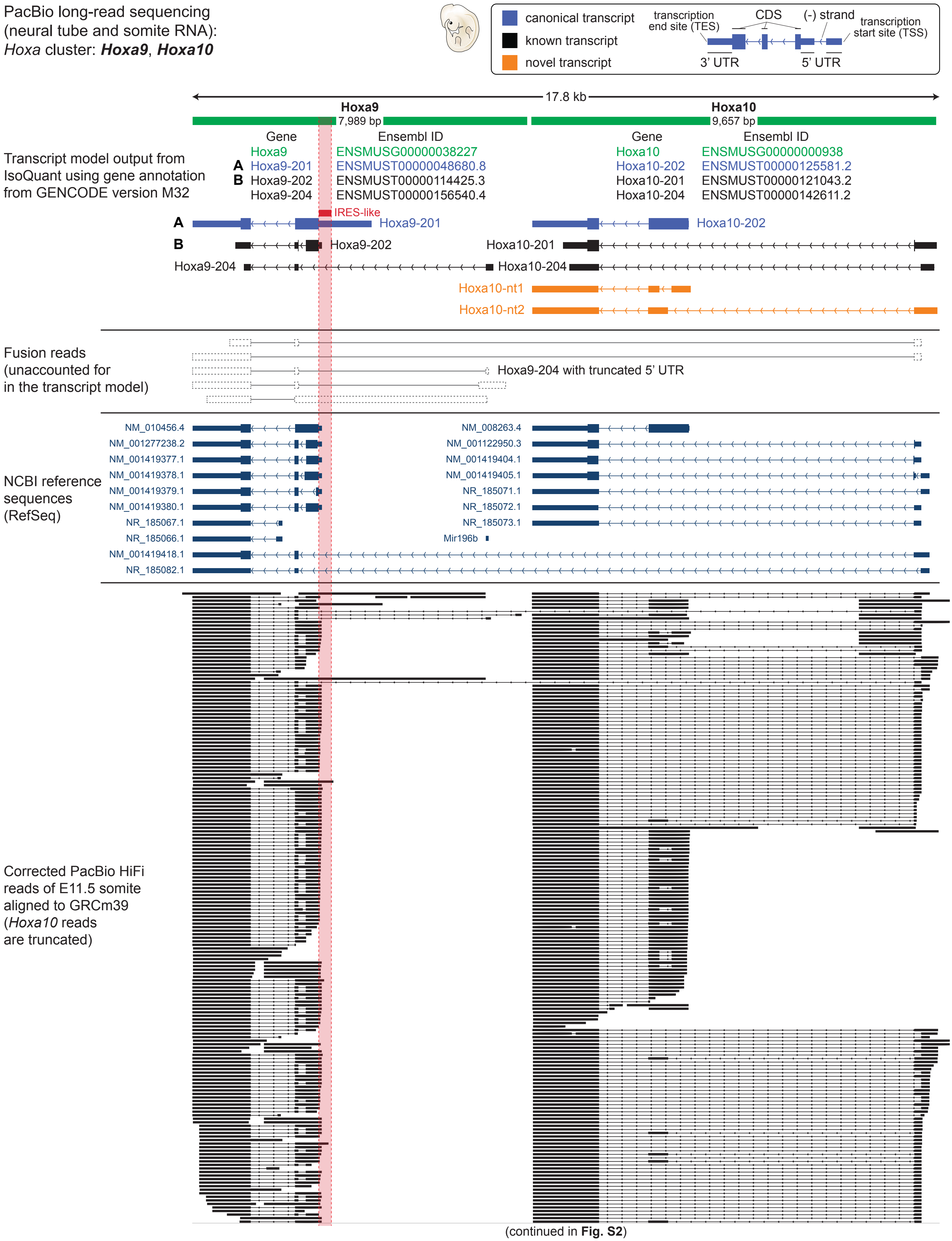

(continued in **Fig. S2**)

Appendix Figure S1. ***Hoxa9 and Hoxa10*** **mRNA expression in mouse embryo somite and neural tube tissues with complete corrected PacBio HiFi reads.**

Illustration of a genome browser snapshot showing the transcript models predicted by IsoQuant, NCBI RefSeq, and corrected HiFi reads from the IsoQuant output around Hoxa9 and Hoxa10 loci (chr6:52,200,050-52,217,850). The 5' UTR IRES-like element tested is indicated by the red box. Despite 5’ UTR truncations, reads of identical intron chains to isoform A were assigned to Hoxa9-201 IRES-containing reference transcript. Similarly, reads of identical intron chains to isoform B but with an extended 3’ UTR were assigned to Hoxa9-202. Only two novel transcripts were detected for the Hoxa10 gene. The previously reported Hoxa9/Mir196b fusion transcript is similar to Hoxa9-204 with a truncated 5’ UTR. Otherwise, there was no novel isoform identified that indicates any fusion transcripts. The full list of the corrected reads is shown in **Appendix Fig. S2**.

Hoxa cluster: ***Hoxa9***, ***Hoxa10***

(continued from **Fig. S1**)

Corrected PacBio HiFi
reads of E11.5 somite
aligned to GRCm39

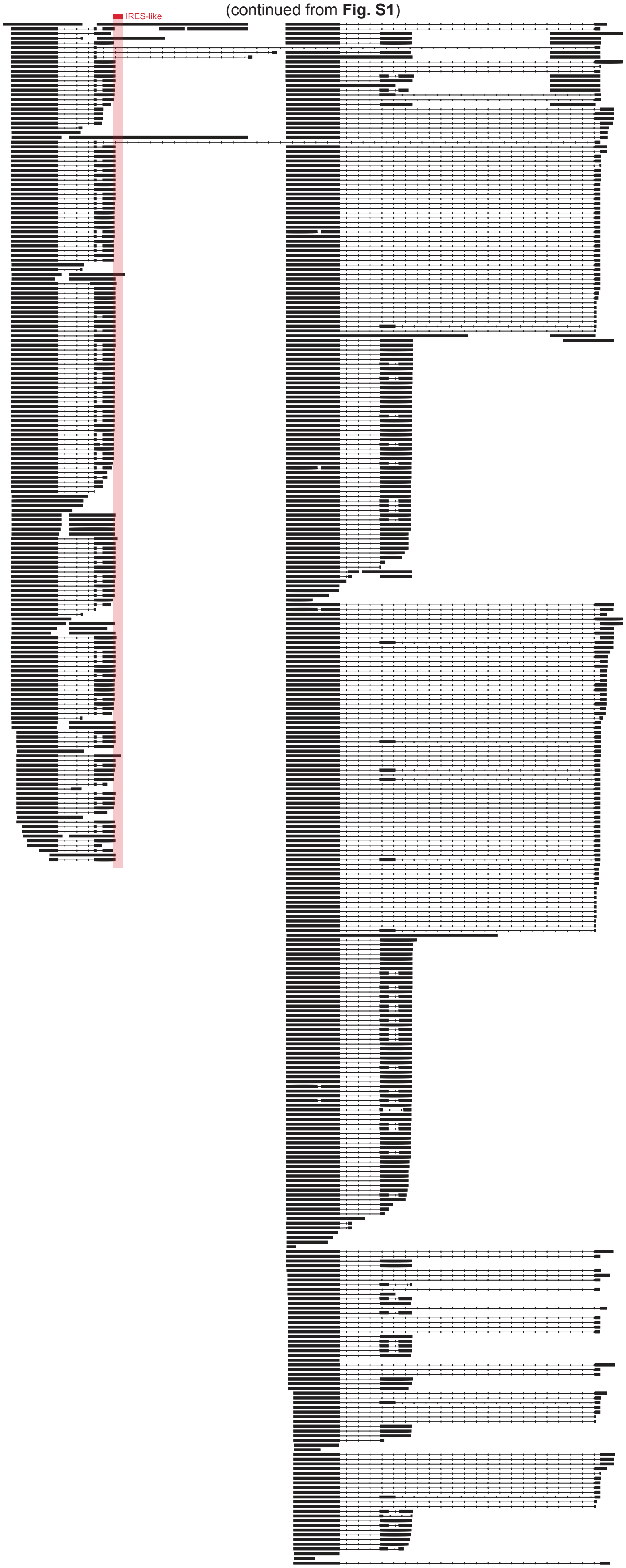

Appendix Figure S2. Complete Hoxa9 and Hoxa10 corrected PacBio HiFi reads (continued reads).

Full display, and continuation from **Appendix Fig. S1**, of corrected PacBio HiFi reads from IsoQuant output that are aligned to Hoxa9 and Hoxa10 locus. Red bar corresponds to the 5’ UTR IRES-like construct tested in this paper.

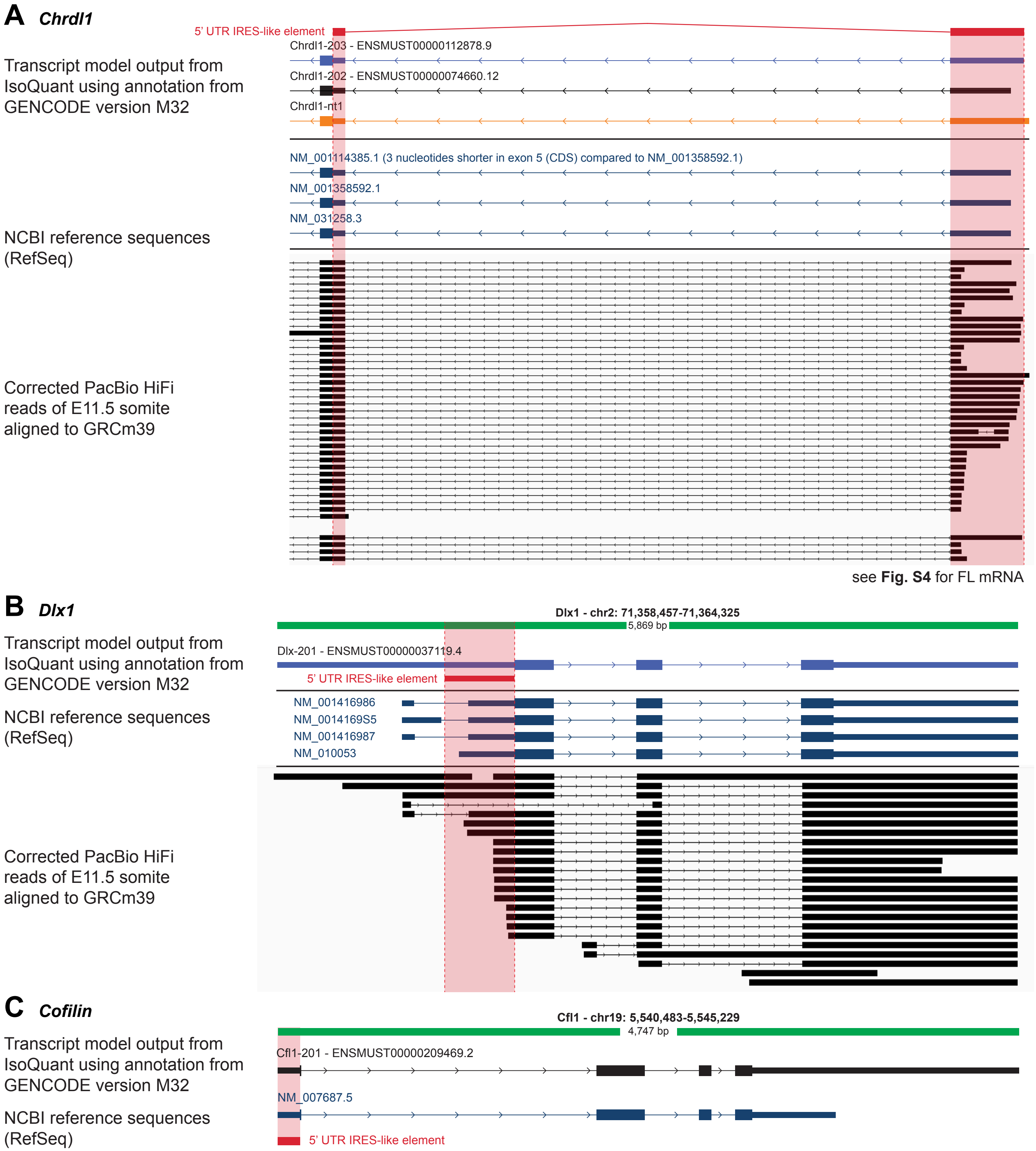
A Chrdl1
5' UTR IRES-like element
Chrdl1-203 - ENSMUST00000112878.9
Chrdl1-202 - ENSMUST00000074660.12
Chrdl1-nt1
Transcript model output from IsoQuant using annotation from GENCODE version M32
NM_001114385.1 (3 nucleotides shorter in exon 5 (CDS) compared to NM_001358592.1)
NM_001358592.1
NM_031258.3
NCBI reference sequences (RefSeq)
Corrected PacBio HiFi reads of E11.5 somite aligned to GRCm39
see Fig. S4 for FL mRNA
B Dlx1
Dlx1 - chr2: 71,358,457-71,364,325
5,869 bp
Dlx-201 - ENSMUST00000037119.4
5' UTR IRES-like element
Transcript model output from IsoQuant using annotation from GENCODE version M32
NM_001416986
NM_0014169S5
NM_001416987
NM_010053
NCBI reference sequences (RefSeq)
Corrected PacBio HiFi reads of E11.5 somite aligned to GRCm39
C Cofilin
Cfl1 - chr19: 5,540,483-5,545,229
4,747 bp
Cfl1-201 - ENSMUST00000209469.2
Transcript model output from IsoQuant using annotation from GENCODE version M32
NM_007687.5
5' UTR IRES-like element
NCBI reference sequences (RefSeq)

Appendix Figure S3. Cellular IRES-like RNA expression in mouse embryo somite and neural tube tissues by PacBio HiFi sequencing.

(**A**) Illustration of a genome browser snapshot showing the transcript models predicted by IsoQuant, NCBI RefSeq, and corrected HiFi reads from the IsoQuant output of the first two exons of *Chrdl1* gene which contains the 5' UTR region. The 5' UTR IRES-like element tested is indicated by the red box. A complete view of the whole gene is shown in **Appendix Fig. S4**.
(**B**) Same genome browser snapshot as in (**A**) for the complete *Dlx1* gene instead. The *Dlx1* canonical transcript was not detected in this dataset.
(**C**) Same genome browser snapshot as in (**A**) for the complete *Cofilin* (*Cfl1*) gene instead, without the corrected reads being displayed, due to the extremely large number of reads that are largely uniform.

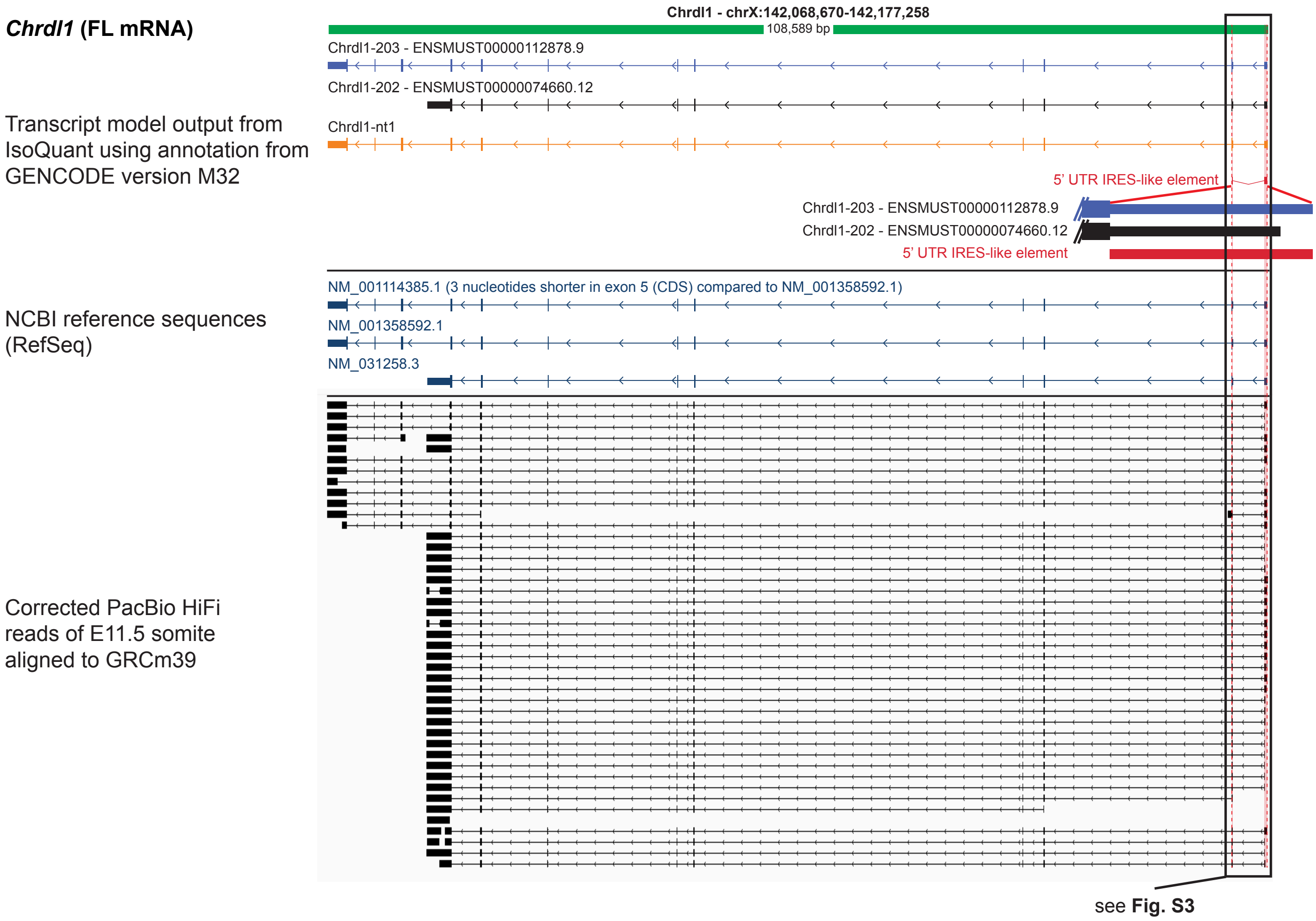
Chrdl1 (FL mRNA)
Chrdl1 - chrX:142,068,670-142,177,258
108,589 bp
Chrdl1-203 - ENSMUST00000112878.9
Chrdl1-202 - ENSMUST00000074660.12
Chrdl1-nt1
Transcript model output from IsoQuant using annotation from GENCODE version M32
5' UTR IRES-like element
Chrdl1-203 - ENSMUST00000112878.9
Chrdl1-202 - ENSMUST00000074660.12
5' UTR IRES-like element
NCBI reference sequences (RefSeq)
NM_001114385.1 (3 nucleotides shorter in exon 5 (CDS) compared to NM_001358592.1)
NM_001358592.1
NM_031258.3
Corrected PacBio HiFi reads of E11.5 somite aligned to GRCm39
see Fig. S3

Appendix Figure S4. A genome browser view showing the complete Chrdl1 locus.
Illustration of a genome browser snapshot showing the transcript models predicted by IsoQuant, NCBI RefSeq, and corrected HiFi reads from IsoQuant output. Only one novel isoform was detected, with a different transcription start site and transcription end site compared to *Chrdl1-203*. Red region corresponds to the 5' UTR IRES-like element tested.

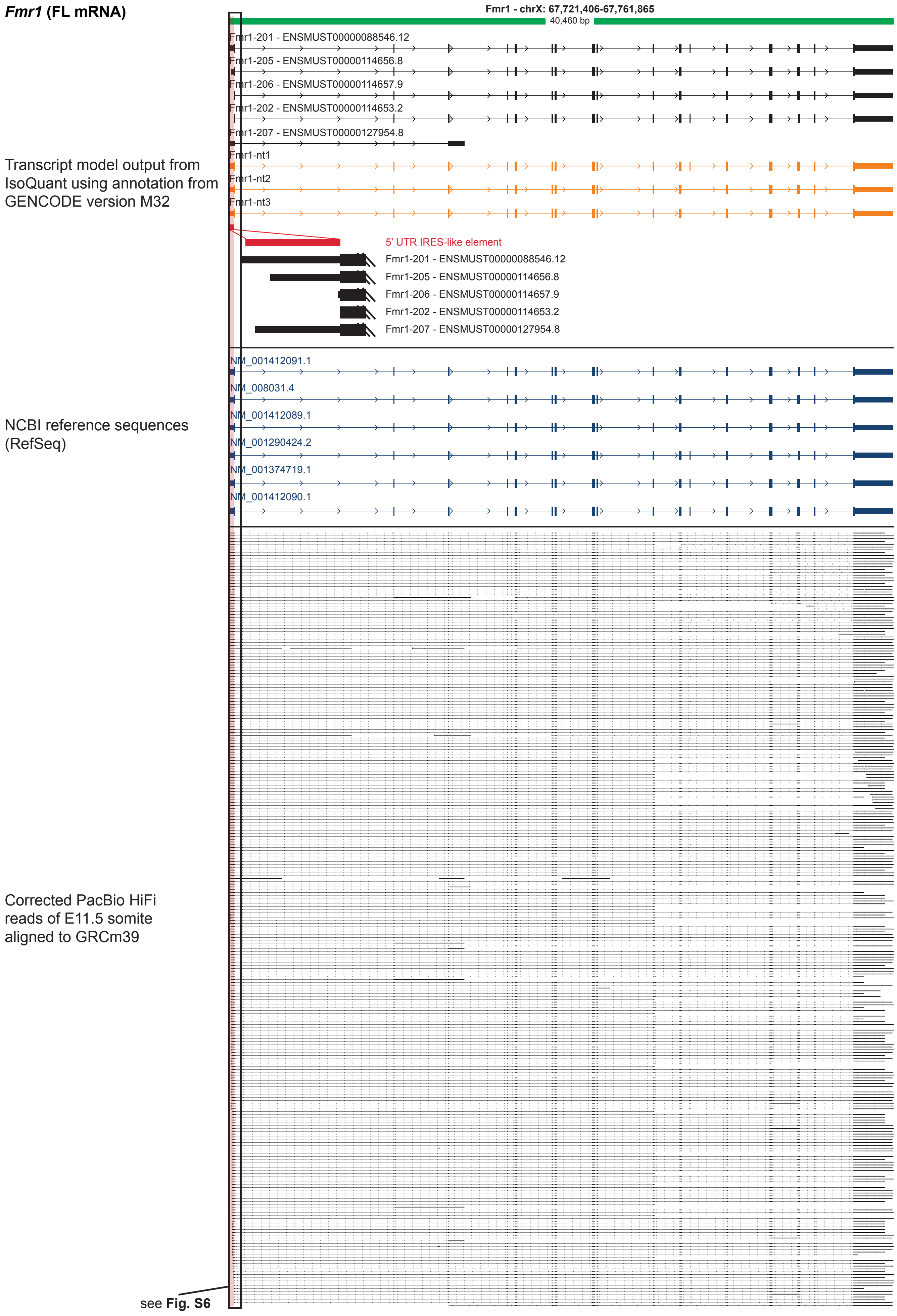
Fmr1 (FL mRNA)
Fmr1 - chrX: 67,721,406-67,761,865
40,460 bp
Fmr1-201 - ENSMUST00000088546.12
Fmr1-205 - ENSMUST00000114656.8
Fmr1-206 - ENSMUST00000114657.9
Fmr1-202 - ENSMUST00000114653.2
Fmr1-207 - ENSMUST00000127954.8
Fmr1-nt1
Fmr1-nt2
Fmr1-nt3
Transcript model output from IsoQuant using annotation from GENCODE version M32
5' UTR IRES-like element
Fmr1-201 - ENSMUST00000088546.12
Fmr1-205 - ENSMUST00000114656.8
Fmr1-206 - ENSMUST00000114657.9
Fmr1-202 - ENSMUST00000114653.2
Fmr1-207 - ENSMUST00000127954.8
NM_001412091.1
NM_008031.4
NM_001412089.1
NM_001290424.2
NM_001374719.1
NM_001412090.1
NCBI reference sequences (RefSeq)
Corrected PacBio HiFi reads of E11.5 somite aligned to GRCm39
see **Fig. S6**

Appendix Figure S5. A genome browser view showing the complete Fmr1 locus.
Illustration of a genome browser snapshot showing the transcript models predicted by IsoQuant, NCBI RefSeq, and corrected HiFi reads from IsoQuant output of the full FMR1 gene. Red bar corresponds to the 5' UTR IRES-like element tested. Zoom-in view of the region in the black box is presented in **Appendix Fig. S6**.

***Fmr1* (5' UTR zoom-in view)**

Transcript model output from IsoQuant using annotation from GENCODE version M32

NCBI reference sequences (RefSeq)

Corrected PacBio HiFi reads of E11.5 somite aligned to GRCm39

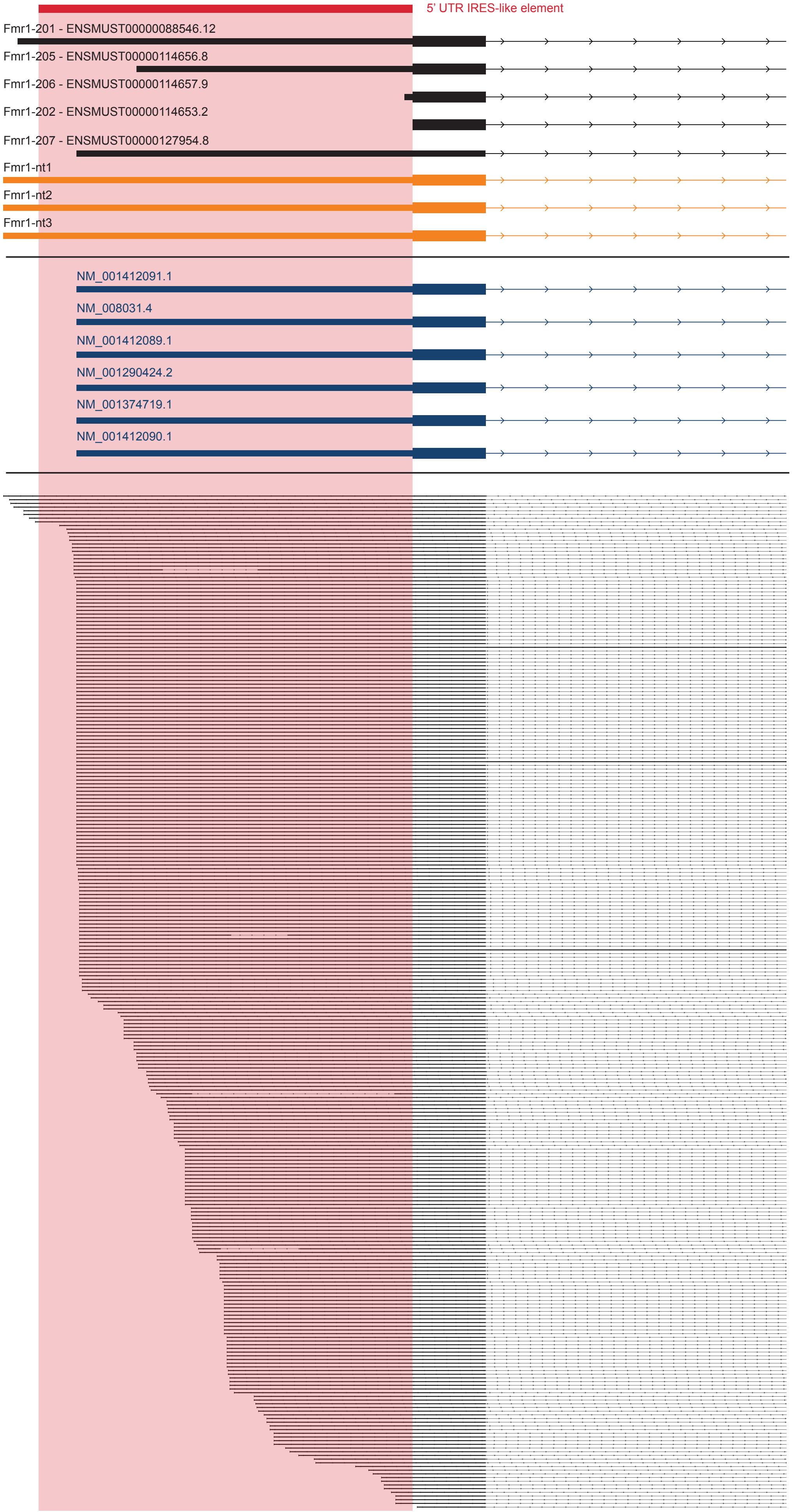

Appendix Figure S6. A genome browser view focusing on 5’ UTR of *Fmr1*.

A zoomed-in view of **Appendix Fig. S5**, highlighting *Fmr1* 5’ UTR region. Red bar corresponds to the 5' UTR IRES-like element tested.

APPENDIX TABLES

Appendix Table S1: Plasmids used in this study.

All plasmids used for mammalian transient transfection are listed in the table.

Appendix Table S1. List of plasmids		
**Plasmid**	**Notes**	**Reference**
Mammalian cells		
**Expression constructs**		
pRF-HCV IRES	pKL033, kindly provided by D. Ruggero	(Yoon et al. 2006)
mRuby3-ZK-spEGFP	pKL477, kindly provided by C. K. Chen	(Chen et al. 2021)
mRuby3-ZK-spEGFP-IRES2(EMCV)	pKL479, kindly provided by C. K. Chen	(Chen et al. 2021)
pcDNA3.1-5'UTR-3xHA-Nluc	pKL401, kindly provided by C. Howard	(Osuna et al. 2017)
pGL3	pKL037, SV40 promoter, Firefly luciferase	Promega
pGL3-HBB	pKL039	(Xue et al. 2015)
pGL3-FLB-stop (Fluc-β-globin)	pKL079	(Leppek et al. 2020)
pGL3-FLB-fusion-HBB5’	pKL082	This study
pCR-Blunt-II-TOPO-HoxA9-3'UTR	pKL090/pK240, kindly provided by K. Fujii	This study
pGL3-NLB-fusion-HBB5’	pMH001	This study
pGL3-Nluc-HBB5’	pMH002	This study
pGL3-NLB-hHBB3'-HBB5'	pMH023	This study
**SV40-promoterless constructs and controls**		
pGL3-SV40minus-NLB-HBB5'-HBB3'	pLL015	This study
pGL3-SV40minus-NLB-empty5'-HBB3'	pLL016	This study
pGL3-SV40minus-NLB-HCV-HBB3'	pLL017	This study
pGL3-SV40minus-NLB-EMCV(IRES2)-HBB3'	pLL018	This study
pGL3-SV40minus-NLB-P4native-HBB3'	pLL019	This study
pGL3-SV40minus-NLB-A3(IRES)-HBB3'	pLL020	This study
pGL3-SV40minus-NLB-A5(IRES)-HBB3'	pLL021	This study
pGL3-SV40minus-NLB-A9(IRES)-HBB3'	pLL022	This study
pGL3-SV40-NLB-empty5'-HBB3'	pLL026	This study
pGL3-SV40-NLB-HCV-HBB3'	pLL027	This study
pGL3-SV40-NLB-EMCV(IRES2)-HBB3'	pLL028	This study
pGL3-SV40-NLB-A3(IRES)-HBB3'	pLL030	This study
pGL3-SV40-NLB-A5(IRES)-HBB3'	pLL031	This study
pGL3-SV40-NLB-A9(IRES)-HBB3'	pLL032	This study
pGL3-SV40minus-NLB-A9(IRES)-M2-HBB3'	pLL036	This study
pGL3-SV40minus-NLB-A9(IRES)-M5-HBB3'	pLL037	This study
pGL3-SV40minus-NLB-A9(IRES)-M12-HBB3'	pLL038	This study
pGL3-SV40minus-NLB-A9(IRES)-M13-HBB3'	pLL039	This study
**circRNA split-EGFP constructs**		

mRuby_ZKSCAN_spEGFP_HBB	pLL008	This study
mRuby_ZKSCAN_spEGFP_HCV	pLL009	This study
mRuby_ZKSCAN_spEGFP_CVB3	pPK008	This study
mRuby_ZKSCAN_spEGFP_P4native	pLL010	This study
mRuby_ZKSCAN_spEGFP_a3(IRES)	pLL011	This study
mRuby_ZKSCAN_spEGFP_a5(IRES)	pLL012	This study
mRuby_ZKSCAN_spEGFP_a9(IRES)	pLL013	This study
mRuby_ZKSCAN_spEGFP_a9(IRES)-M2	pLL040	This study
mRuby_ZKSCAN_spEGFP_a9(IRES)-M5	pLL041	This study
mRuby_ZKSCAN_spEGFP_a9(IRES)-M12	pLL042	This study
mRuby_ZKSCAN_spEGFP_a9(IRES)-M13	pLL043	This study
mRuby_ZKSCAN_spEGFP_invHBB	pPK009	This study
mRuby_ZKSCAN_spEGFP_invHCV	pPK010	This study
mRuby_ZKSCAN_spEGFP_invEMCV	pPK011	This study
mRuby_ZKSCAN_spEGFP_invA9	pPK012	This study
mRuby_ZKSCAN_spEGFP_invA5	pPK013	This study
mRuby_ZKSCAN_spEGFP_invCVB3	pPK015	This study
mRuby3_ZKSCAN_spEGFP_Chrdl1(IRES)	pPK026	This study
mRuby3_ZKSCAN_spEGFP_Dlx1(IRES)	pPK027	This study
mRuby3_ZKSCAN_spEGFP_Gdf5(IRES)	pPK028	This study
mRuby3_ZKSCAN_spEGFP_Sema3a(IRES)	pPK029	This study
mRuby3_ZKSCAN_spEGFP_Zfx(IRES)	pPK030	This study
mRuby3_ZKSCAN_spEGFP_c-Myc(IRES)	pPK031	This study
mRuby3_ZKSCAN_spEGFP_CACNA1A(IRES)	pPK032	This study
mRuby3_ZKSCAN_spEGFP_Cofilin(IRES)	pPK033	This study
mRuby3_ZKSCAN_spEGFP_FMR1(IRES)	pPK034	This study
mRuby3_ZKSCAN_spEGFP_c-Myc(IRES) Inv	pPK037	This study
mRuby3_ZKSCAN_spEGFP_Chrdl1(IRES) Inv	pPK038	This study
mRuby3_ZKSCAN_spEGFP_Dlx1(IRES) Inv	pPK039	This study
mRuby3_ZKSCAN_spEGFP_FMR1(IRES) Inv	pPK040	This study
mRuby3_ZKSCAN_spEGFP_Sema3a(IRES) Inv	pPK041	This study
mRuby3_ZKSCAN_spEGFP_Bcl2(IRES)	pPK042	This study
mRuby3_ZKSCAN_spEGFP_Bcl2(IRES) Inv	pPK043	This study
mRuby3_ZKSCAN_spEGFP_Cofilin(IRES) Inv	pPK044	This study

Appendix Table S2: DNA Oligonucleotides used in this study.

All DNA oligonucleotides used for cloning, RT-PCR, and RT-qPCR are listed in the table. F, forward primer; R, reverse primer.

Appendix Table S2. DNA oligonucleotides

Name	Sequence	Description
	qPCR primer	
KL050	TGGAGAATAACTTCTTCGTGGA	Rluc qPCR F
KL051	TTGGACGACGAACTTCACC	Rluc qPCR R
KL052	AAGAGATACGCCCTGGTTC	Fluc qPCR F
KL053	TTGTATTCAGCCCATATCGTTTC	Fluc qPCR R
KL056	GCCAACCGTGAAAAGATGAC	mouse β-*actin* F
KL057	CATCACAATGCCTGTGGTAC	mouse β-*actin* R
KL075	GAAGGCTCATGGCAAGAAGG	rabbit β-*globin* qPCR F
KL076	ATGATGAGACAGCACAATAACCAG	rabbit β-*globin* qPCR R
KL412	CATGGCTGCAACACTTACACAGCA	mouse *Nupl1* qPCR F
KL413	ATTGCAAGCCAGTGCCAATACCTG	mouse *Nupl1* qPCR R
KL109	AAAAACAACCCAGCGAAGGC	mouse *Hoxa9* qPCR F (CDS2 ampl.)
KL110	ATCGCTTCTTCCGAGTGGAG	mouse *Hoxa9* qPCR R (CDS2 ampl.)
KL585	CCGTATGAAGGTCTGAGCGG	Nanoluc qPCR F
KL586	CAGTGTGCCATAGTGCAGGA	Nanoluc qPCR R
KL596	CTTCGTTGGCCACAATTAAAACAAACCAG	*a9* IRES primer (mouse) F (IRES1)
KL597	GCCCAGCAGGAAGGAGTC	*a9* CDS primer (mouse) R (IRES1&4)
JS98	GCGCGATCCCTTTGCATAAA	*Hoxa9* IRES2 amplicon F
JS99	CCGACCCGCCGAAATTATGA	*Hoxa9* IRES2 and IRES3 amplicons R
JS104	TGCAAAACACCGGGCCATTA	*Hoxa9* IRES3 and IRES4 amplicons F
NG771	TGGTGCCACCAAGTTGTTACATG	85 nt 5' UTR *Hoxa9* amplicon F
NG761	TGCAGTAGCCCGCGCCTG	85 nt 5' UTR *Hoxa9* amplicon R
NG808	GCCCTGGGCAACTACTATGT	*Hoxa9* CDS 5' amplicon F
NG809	GGACTGAAGTCGGGGTGTTC	*Hoxa9* CDS 5' amplicon R
	SV40-promoterless plasmids and +SV40 controls	
LL042	CGCGTGCTAGCCCGGGCTCGAGATCTAAGCTTacatttgcttctgacacaac	SV40minus-oligo-1 F
LL043	gttgtgtcagaagcaaatgtAAGCTTAGATCTCGAGCCCGGGCTAGCACGCG	SV40minus-oligo-1 R
LL044	CGCGTGCTAGCCCGGGCTCGAGATCTGCCACCATGGCCGTTTACCC	SV40minus-oligo-2 F
LL045	GGGTAAACGGCCATGGTGGCAGATCTCGAGCCCGGGCTAGCACGCG	SV40minus-oligo-2 R
LL046	CTAGCCCGGGCTCGAGATCTTTGGGGGCGACACTCC	SV40minus-HCV Gibson F
LL047	TCGTATGGGTAAACGGCCATGGTGGCTTTACCAACGAATTC	SV40minus-HCV Gibson R
LL048	CTAGCCCGGGCTCGAGATCTcccctctccctcccc	SV40minus-EMCV Gibson F
LL049	TCGTATGGGTAAACGGCCATGATggttgtggccatattatcatcgt	SV40minus-EMCV Gibson R
LL052	CTAGCCCGGGCTCGAGATCTtggcggcggagtgtc	SV40minus-A3 Gibson F
LL053	TCGTATGGGTAAACGGCCATggcgcgatgtttcacgatc	SV40minus-A3 Gibson R
LL054	CTAGCCCGGGCTCGAGATCTatcaggcaggatttacgactg	SV40minus-A5 Gibson F
LL055	TCGTATGGGTAAACGGCCATggtgcttgatttgtggctcg	SV40minus-A5 Gibson R
LL056	CTAGCCCGGGCTCGAGATCTttgatcttttaatcttcgttggcca	SV40minus-A9 Gibson F
LL057	TCGTATGGGTAAACGGCCATggtgcagtagcccgc	SV40minus-A9 Gibson R
LL058	TAGGCTTTTGCAAAAAGCTTTTGGGGGCGACACTCC	SV40-HCV Gibson F
LL059	TAGGCTTTTGCAAAAAGCTTcccctctccctcccc	SV40-EMCV Gibson F
LL061	TAGGCTTTTGCAAAAAGCTTtggcggcggagtgtc	SV40-A3 Gibson F
LL062	TAGGCTTTTGCAAAAAGCTTatcaggcaggatttacgactg	SV40-A5 Gibson F

LL063	TAGGCTTTTGCAAAAAGCTTttgatcttttaatcttcgttggcca	SV40-A9 Gibson F
LL064	GAGGCCTAGGCTTTTGCAAAAAGCTTGCCACCATGGCCGTTTACCC	SV40-oligo-3 F
LL065	GGGTAAACGGCCATGGTGGCAAGCTTTTTGCAAAAGCCTAGGCCTC	SV40-oligo-3 R
LL068	CTAGCCCGGGCTCGAGATCTAAGCTTacatttgcttctgacacaa	SV40minus-HBB Gibson F
LL069	TCGTATGGGTAAACGGCCATGGTGGCGATATCggtgtctgtttga	SV40minus-HBB Gibson R
LL070	CGCGTTATTGTTCTGCCGGGCGTTGACGTGACGCG	SV40minus-A9-M2 Gibson F
LL071	CGCGTTATTGTTCTGCCGGGCGGACACTATTCGCG	SV40minus-A9-M5 Gibson F
LL072	CGCGTTATTGTTCTGCCGGGCGTTCACGTGACGCG	SV40minus-A9-M12 Gibson F
LL073	CGCGTTATTGTTCTGCCGGGCGGACACGTGTGCCG	SV40minus-A9-M13 Gibson F
LL074	CGCGTCACGTCAACGCCCGGCAGAACAATAACGCG	SV40minus-A9-M2 Gibson R
LL075	CGCGAATAGTGTCCGCCCGGCAGAACAATAACGCG	SV40minus-A9-M5 Gibson R
LL076	CGCGTCACGTGAACGCCCGGCAGAACAATAACGCG	SV40minus-A9-M12 Gibson R
LL077	CGGCACACGTGTCCGCCCGGCAGAACAATAACGCG	SV40minus-A9-M13 Gibson R
	Cloning of mRuby-ZSCAN-splitEGFP reporter plasmids	
LL017	ACGAGCTGTACAAGTAAGATacatttgcttctgacacaact	ZK-spEGFP HBB F
LL018	TCCTCGCCCTTGCTCACCATggtgtctgtttgaggttg	ZK-spEGFP HBB R
LL019	ACGAGCTGTACAAGTAAGATTTGGGGGCGACACT	ZK-spEGFP HCV F
LL020	TCCTCGCCCTTGCTCACCATGGTGGCTTTACCAACGAAT	ZK-spEGFP HCV R
LL021	ACGAGCTGTACAAGTAAGATcgcgttattgttctgccg	ZK-spEGFP P4native F
LL022	TCCTCGCCCTTGCTCACCATggtgcagtagcccgcg	ZK-spEGFP P4native R
LL023	ACGAGCTGTACAAGTAAGATtggcggcggagt	ZK-spEGFP A3(IRES) F
LL024	TCCTCGCCCTTGCTCACCATggcgcgatgtttcacgatcg	ZK-spEGFP A3(IRES) R
LL025	ACGAGCTGTACAAGTAAGATatcaggcaggatttacgac	ZK-spEGFP A5(IRES) F
LL026	TCCTCGCCCTTGCTCACCATggtgcttgatttgtggctcg	ZK-spEGFP A5(IRES) R
LL027	ACGAGCTGTACAAGTAAGATttgatcttttaatcttcgttgg	ZK-spEGFP A9(IRES) F
LL028	TCCTCGCCCTTGCTCACCATggtgcagtagcccgcg	ZK-spEGFP A9(IRES) R
PK100	TTAAAACAGCCTGTGGGTTGATCCCACCCACAGGCCCATTGGGCGCTAGCACTCTGGTAT	ZK-spEGFP CVB3_F
PK101	TGGGGGAGGGGGTATAAAACAGGCGCACAAAGGTACCGTGATACCAGAGTGCTAGCGCCC	ZK-spEGFP CVB3_R
PK102	GTTTTATACCCCCTCCCCCAACTGTAACTTAGAAGTAACACACACCGATCAACAGTCAGC	ZK-spEGFP CVB3_F
PK103	GTAACAGAAGTGCTTGATCAAAACGTGGCTGGTGTGCCACGCTGACTGTTGATCGGTGTG	ZK-spEGFP CVB3_R
PK104	TGATCAAGCACTTCTGTTACCCCGGACTGAGTATCAATAGACTGCTCACGCGGTTGAAGG	ZK-spEGFP CVB3_F
PK105	AGGTTTTTCGAAGTAGTTGGCCGGATAACGAACGCTTTCTCCTTCAACCGCGTGAGCAGT	ZK-spEGFP CVB3_R
PK106	CCAACTACTTCGAAAAACCTAGTAACACCGTGGAAGTTGCAGAGTGTTTCGCTCAGCACT	ZK-spEGFP CVB3_F
PK107	GGGGAATGCGGTGACTCATCGACCTGATCTACACTGGGGTAGTGCTGAGCGAAACACTCT	ZK-spEGFP CVB3_R
PK108	GATGAGTCACCGCATTCCCCACGGGCGACCGTGGCGGTGGCTGCGTTGGCGGCCTGCCCA	ZK-spEGFP CVB3_F
PK109	CGCACCATGTCTGTATTAGAGCGTCCCATGGGTTTCCCCATGGGCAGGCCGCCAACGCAG	ZK-spEGFP CVB3_R
PK110	TCTAATACAGACATGGTGCGAAGAGTCTATTGAGCTAGTTGGTAGTCCTCCGGCCCCTGA	ZK-spEGFP CVB3_F
PK111	GGCTTGAGGGTGTGTGCTCCGCAGTTAGGATTAGCCGCATTCAGGGGCCGGAGGACTACC	ZK-spEGFP CVB3_R
PK112	GGAGCACACACCCTCAAGCCAGAGGGCAGTGTGTCGTAACGGGCAACTCTGCAGCGGAAC	ZK-spEGFP CVB3_F
PK113	GTATAGGAATAAAATGAAACACGGACACCCAAAGTAGTCGGTTCCGCTGCAGAGTTGCCC	ZK-spEGFP CVB3_R

PK114	GTTTCATTTTATTCCTATACTGGCTGCTTATGGTGACAATTGAGAGATCGTTACCATATA	ZK-spEGFP CVB3_F
PK115	ATAATAGCTCTATTAGTCACCGGATGGCCAATCCAATAGCTATATGGTAACGATCTCTCA	ZK-spEGFP CVB3_R
PK116	GTGACTAATAGAGCTATTATATATCCCTTTGTTGGGTTTATACCACTTAGCTTGAAAGAG	ZK-spEGFP CVB3_F
PK117	GGTTTGCTGTATTCAACTTAACAATGAATTGTAATGTTTTAACCTCTTTCAAGCTAAGTGGTA	ZK-spEGFP CVB3_R
PK118	CGAGCTGTACAAGTAAGATTTAAAACAGCCTGTGGGTTGATCC	ZK-spEGFP GACVB3_F
PK119	CCCTTGCTCACCATGATGGTTTGCTGTATTCAACTTAACAATGAATTGTAATG	ZK-spEGFP GACVB3_R
PK130	CGAGCTGTACAAGTAAGATggtgtctgtttgaggttgctagtga	ZK-spEGFP invHBB F
PK131	CCCTTGCTCACCATacatttgcttctgacacaactgtgt	ZK-spEGFP invHBB R
PK132	CGAGCTGTACAAGTAAGATGGTGGCTTTACCAACGAATTCG	ZK-spEGFP invHCV F
PK133	CCCTTGCTCACCATTTGGGGGCGACACTCCAC	ZK-spEGFP invHCV R
PK134	CGAGCTGTACAAGTAAGATggttgtggccatattatcatcgtgt	ZK-spEGFP invEMCV F
PK135	CCCTTGCTCACCATGATcccctctccctccccccc	ZK-spEGFP invEMCV R
PK136	CGAGCTGTACAAGTAAGATggtgcagtagcccgcg	ZK-spEGFP invA9 F
PK137	CCCTTGCTCACCATttgatcttttaatcttcgttggccacaattaaaac	ZK-spEGFP invA9 R
PK138	CCCTTGCTCACCATttgatcttttaatcttcgttggccacaattaaaac	ZK-spEGFP invA5 F
PK139	CCCTTGCTCACCATatcaggcaggatttacgactggac	ZK-spEGFP invA5 R
PK142	CGAGCTGTACAAGTAAGATGGTTTGCTGTATTCAACTTAACAATGAATTGTAATG	ZK-spEGFP invCVB3 F
PK143	CCCTTGCTCACCATGATTTAAAACAGCCTGTGGGTTGATCC	ZK-spEGFP invCVB3 R
PK175	ACGAGCTGTACAAGTAAGATACTAGAACTCGCTGTAGTAATTCCAGC	ZK-spEGFP cMyc_F
PK176	TCCTCGCCCTTGCTCACCATggTCGCGGGAGGCTGC	ZK-spEGFP cMyc_R
PK177	ACGAGCTGTACAAGTAAGATCAGGCGTTGGGGGCG	ZK-spEGFP Dlx1_F
PK178	TCCTCGCCCTTGCTCACCATggCTCTTCTCGCGGGGTCTG	ZK-spEGFP Dlx1_R
PK179	ACGAGCTGTACAAGTAAGATGAAAGTCATGGCTTTTGGGATTCTGAA	ZK-spEGFP CACNA1A_F
PK180	TCCTCGCCCTTGCTCACCATggACGGTGAGGTCCGTGGAC	ZK-spEGFP CACNA1A_R
PK181	ACGAGCTGTACAAGTAAGATGCCGGAAGGCCGCC	ZK-spEGFP Cofilin_F
PK182	TCCTCGCCCTTGCTCACCATggGTTTCCGGAAACGAAAGGGAGAC	ZK-spEGFP Cofilin_R
PK183	ACGAGCTGTACAAGTAAGATGTGTGGTGGGGGCGC	ZK-spEGFP Chrdl1_F
PK184	TCCTCGCCCTTGCTCACCATggCTTCTACTTTTTTCTCCTTCGAGCTACTG	ZK-spEGFP Chrdl1_R
PK187	ACGAGCTGTACAAGTAAGATTTCAAGCCCTCAGTCAGTTGTGC	ZK-spEGFP Gdf5_F
PK188	TCCTCGCCCTTGCTCACCATggCCTCTGGCCAGCCGC	ZK-spEGFP Gdf_R
PK189	ACGAGCTGTACAAGTAAGATGTGACAAGAGGGAAGGGGAGT	ZK-spEGFP Sema3a_F
PK190	TCCTCGCCCTTGCTCACCATggGCTGCAGACGCTGGAGG	ZK-spEGFP Sema3a_R
PK191	ACGAGCTGTACAAGTAAGATACGGAAACTCGGGCCG	ZK-spEGFP Zfx_F
PK192	TCCTCGCCCTTGCTCACCATggGGCCTTTAAATTCTCATCAGCCAGAAC	ZK-spEGFP Zfx_R
PK193	ACGAGCTGTACAAGTAAGATAGGAGGCGCAGCGG	ZK-spEGFP FMR1_F
PK194	TCCTCGCCCTTGCTCACCATggCTTCTCGTCCGTCCTCGC	ZK-spEGFP FMR1_R
PK215	ACGAGCTGTACAAGTAAGATCTTCTACTTTTTTCTCCTTCGAGCTACTG	ZK-spEGFP Chrdl1_Fo
PK216	TCCTCGCCCTTGCTCACCATGggTGTGGTGGGGGCGC	ZK-spEGFP Chrdl1_Re
PK217	ACGAGCTGTACAAGTAAGATTCGCGGGAGGCTGC	ZK-spEGFP CMyc_F
PK218	TCCTCGCCCTTGCTCACCATggACTAGAACTCGCTGTAGTAATTCCAGC	ZK-spEGFP CMyc_R
PK219	ACGAGCTGTACAAGTAAGATCTCTTCTCGCGGGGTCTG	ZK-spEGFP Dlx1_F
PK220	TCCTCGCCCTTGCTCACCATggCAGGCGTTGGGGGCG	ZK-spEGFP Dlx1_R
PK221	ACGAGCTGTACAAGTAAGATCTTCTCGTCCGTCCTCGC	ZK-spEGFP FMR_F
PK222	TCCTCGCCCTTGCTCACCATggAGGAGGCGCAGCGG	ZK-spEGFP FMR_R
PK223	ACGAGCTGTACAAGTAAGATGCTGCAGACGCTGGAGG	ZK-spEGFP Sema3a_F
PK224	TCCTCGCCCTTGCTCACCATGggTGACAAGAGGGAAGGGGAGT	ZK-spEGFP Sema3a_R

PK268	ACGAGCTGTACAAGTAAGATGCGCCCGCCCCT	ZK-spEGFP Bcl2_F
PK269	TCCTCGCCCTTGCTCACCATggCCTTCCCAGAGGAAAAGCAACG	ZK-spEGFP Bcl2_R
PK270	ACGAGCTGTACAAGTAAGATCCTTCCCAGAGGAAAAGCAACG	ZK-spEGFP Bcl2 inv_F
PK271	TCCTCGCCCTTGCTCACCATggGCGCCCGCCCCT	ZK-spEGFP Bcl2 inv_R
PK272	ACGAGCTGTACAAGTAAGATGTTTCCGGAAACGAAAGGGAGAC	ZK-spEGFP Cofilin inv_F
PK273	TCCTCGCCCTTGCTCACCATGGGCCGGAAGGCCGCC	ZK-spEGFP Cofilin inv_R
	qPCR Primer for linear/circRNA quantification	
LL034	Atgaaggtggtcatggaagg	mRuby3-RTqPCR-F
LL035	Tccctcgatgactttgatcc	mRuby3-RTqPCR-R
LL036	CAGAAGAACGGCATCAAGGT	Circ-junction-RTqPCR-F
LL037	GGGGGTGTTCTGCTGGTAG	Circ-junction-RTqPCR-R
	Cloning of mRuby-ZSCAN-splitEGFP mutation plasmids	
LL070	CGCGTTATTGTTCTGCCGGGCGTTGACGTGACGCG	SV40minus-A9-M2 Gibs F
LL074	CGCGTCACGTCAACGCCCGGCAGAACAATAACGCG	SV40minus-A9-M2 Gibs R
LL071	CGCGTTATTGTTCTGCCGGGCGGACACTATTCGCG	SV40minus-A9-M5 Gibs F
LL075	CGCGAATAGTGTCCGCCCGGCAGAACAATAACGCG	SV40minus-A9-M5 Gibs R
LL072	CGCGTTATTGTTCTGCCGGGCGTTCACGTGACGCG	SV40minus-A9-M12 Gibs F
LL076	CGCGTCACGTGAACGCCCGGCAGAACAATAACGCG	SV40minus-A9-M12 Gibs R
LL073	CGCGTTATTGTTCTGCCGGGCGGACACGTGTGCCG	SV40minus-A9-M13 Gibs F
LL077	CGGCACACGTGTCCGCCCGGCAGAACAATAACGCG	SV40minus-A9-M13 Gibs R
	Cloning of pGL3-Nluc and -NLB plasmids	
MH001	aacctcaaacagacaccGATATCGCCACCATGGCCGTTTACCCATACGAT	pMH001/002 Nluc insert part 1 F
MH002	ctggacagatgcacGCCCATCTGCTGGCCGCCGGTCATGCTGGCCATggtgga tGATCCGAGCTCGGTACCCGCCAGAATGCGTTCG	pMH001 Nluc insert part 2 R
MH003	GCCGGCCGCCCCGACTCTAGAATTACGCCAGAATGCGTTCG	pMH002 Nluc insert part 2 R
MH008	GCGAACGCATTCTGGCGTAAGCTCGCTTTCTTGCTGTCC	pMH023 HBB3’ insert F
MH009	GCCGGCCGCCCCGACTCTAGATTGCAATGAAAATAAATGTTTTTTATTAGGCA GAATCC	pMH023 HBB’3 insert R
MH055	CGAACGCATTCTGGCGGGTACCGAGCTCGGATCatccaccATGGCCAGCATGA CC	pMH001 Nluc insert part 1 R
MH056	GGTACCCGCCAGAATGCGTTCG	pMH001 Nluc insert part 2 F
