# Supplementary material for: A versatile toolbox for determining IRES activity in cells and embryonic tissues
Source: EMBO J. 2025 Mar 13;44(9):2695–724. doi: 10.1038/s44318-025-00404-5 (PMC12048685; doi:10.1038/s44318-025-00404-5)
Supplement: Supplementary file 10 — Expanded View Figures [file 44318_2025_404_MOESM10_ESM.pdf]

Expanded View Figures

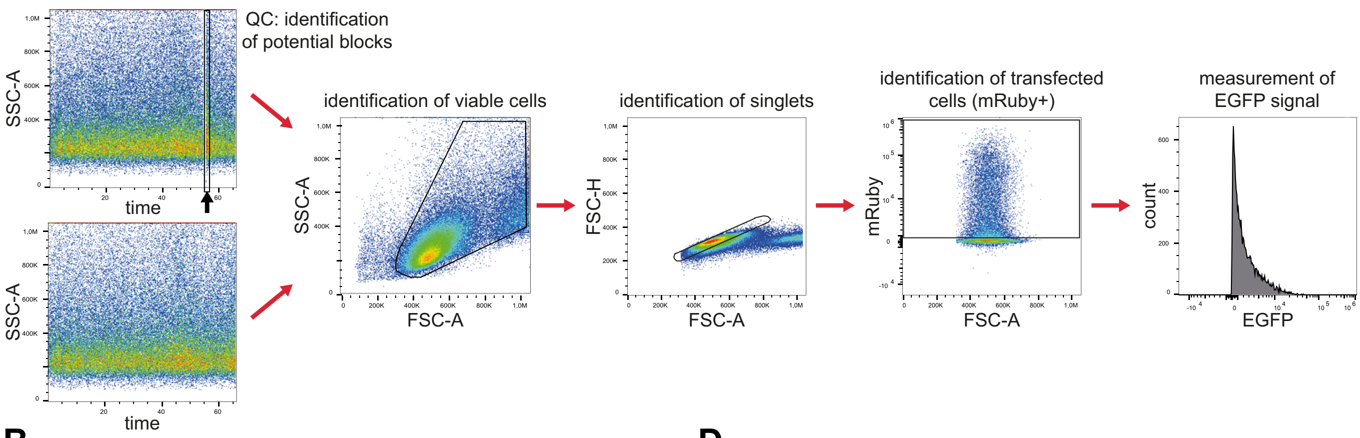

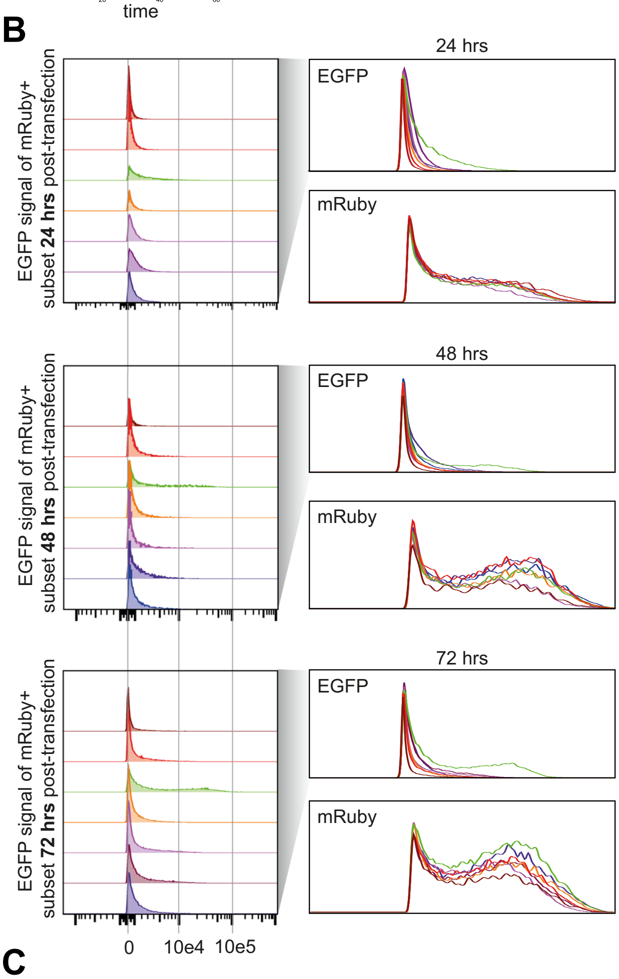

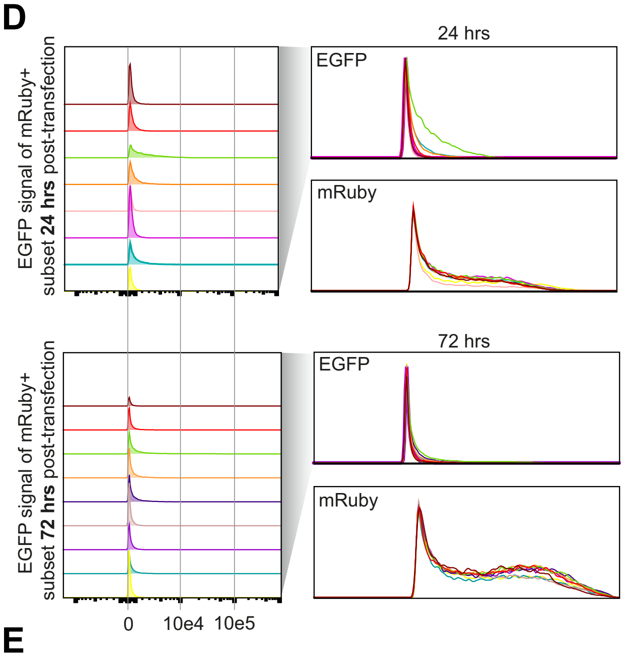

E

	sample	subset	count 24 h	count 72 h
	empty	mRuby+	10901	5004
	hHBB	mRuby+	9132	14267
	HCV	mRuby+	11874	23513
	P4-native	mRuby+	10179	22299
	a9 IRES	mRuby+	6988	22523
	a9 M2	mRuby+	6275	22757
	a9 M5	mRuby+	14754	15445
	a9 M12	mRuby+	10345	14390
	a9 M13	mRuby+	7168	25013

C

	sample	subset	count 24 h	count 48 h	count 72 h
	empty	mRuby+	10725	1633	6690
	hHBB	mRuby+	15171	4897	12730
	HCV	mRuby+	11345	4293	16813
	P4-native	mRuby+	6763	5192	14161
	a3 IRES	mRuby+	11330	4574	16161
	a5 IRES	mRuby+	13278	5245	15585
	a9 IRES	mRuby+	12647	4805	14541

© The Author(s)

Figure EV1. FACS Gating Scheme for circRNA plasmid-derived EGFP assays.

(**A**) Schematic overview of the raw FACS data processing pipeline including: initial quality control (to exclude low-quality data derived from cells measured after a clogging event of the FACS machine), identification of viable cells and singlets, identification of transfected cells (based on the positive mRuby signal), and final measurement of the EGFP signal of the mRuby+ subfraction. (**B**) EGFP signals of the mRuby+ subfraction of cells representatively shown as histograms for one experiment for each observed time point (left panels) and overlayed histograms of the EGFP and mRuby signal of the mRuby+ subfraction of cells (right panels), colorized according to the tested sample sequences according to Fig. EV1C. (**C**) Cell numbers of the mRuby+ cell fractions of the indicated samples shown for one representative experiment per time point. (**D**) EGFP signals of the mRuby+ subfraction of cells representatively shown as histograms for one experiment for each observed time point (left panels) and overlayed histograms of the EGFP and mRuby signal of the mRuby+ subfraction of cells (right panels), colorized according to the tested sample sequences according to Fig. EV1E. (**E**) Cell numbers of the mRuby+ cell fractions of the indicated samples shown for one representative experiment per time point.

© The Author(s)

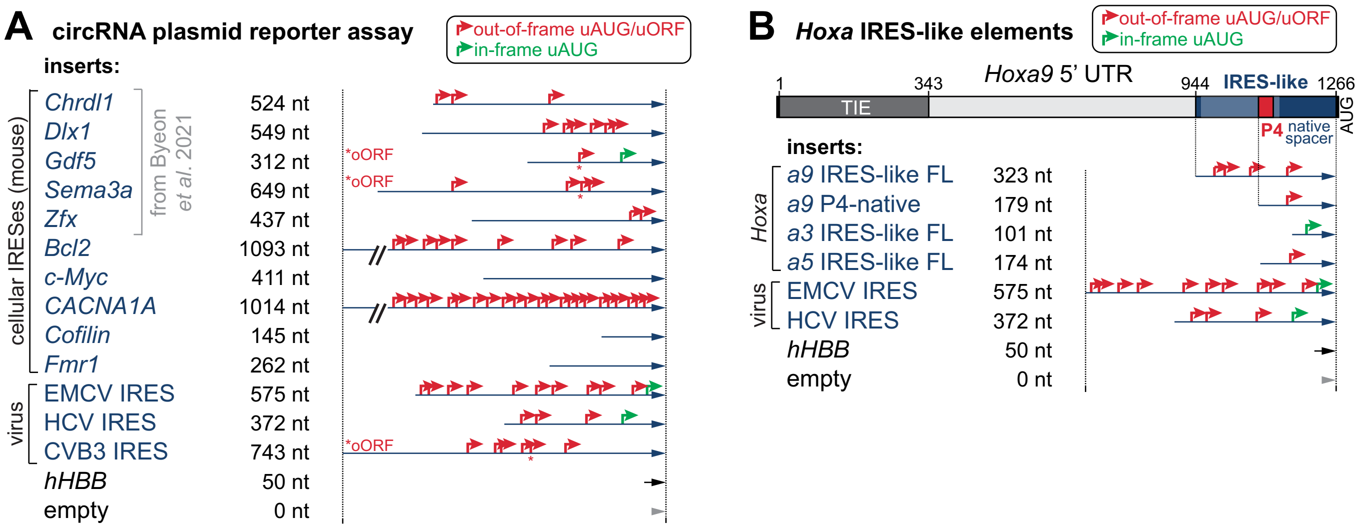

Figure EV2. Schematic of IRES-like elements for circRNA plasmid-derived EGFP assays and annotated uAUGs.

(**A**) Schematic of the circRNA reporter assay based on the mRuby-ZKSCAN-split-EGFP plasmid for the screening of IRES-like activity of the different tested insert sequences, with annotated out-of-frame upstream (u)AUGs/uORFs (red) and overlapping ORFs (oORF, asterisks), and in-frame uAUGs (green) mapped onto the IRES-like sequences (adapted from Fig. 1A). (**B**) Schematic of different tested insert sequences as in (**A**) for the *Hoxa* cluster IRES-like elements, with annotated out-of-frame uAUGs/uORFs (red), and in-frame uAUGs (green) mapped onto the IRES-like sequences (adapted from Fig. 2A).

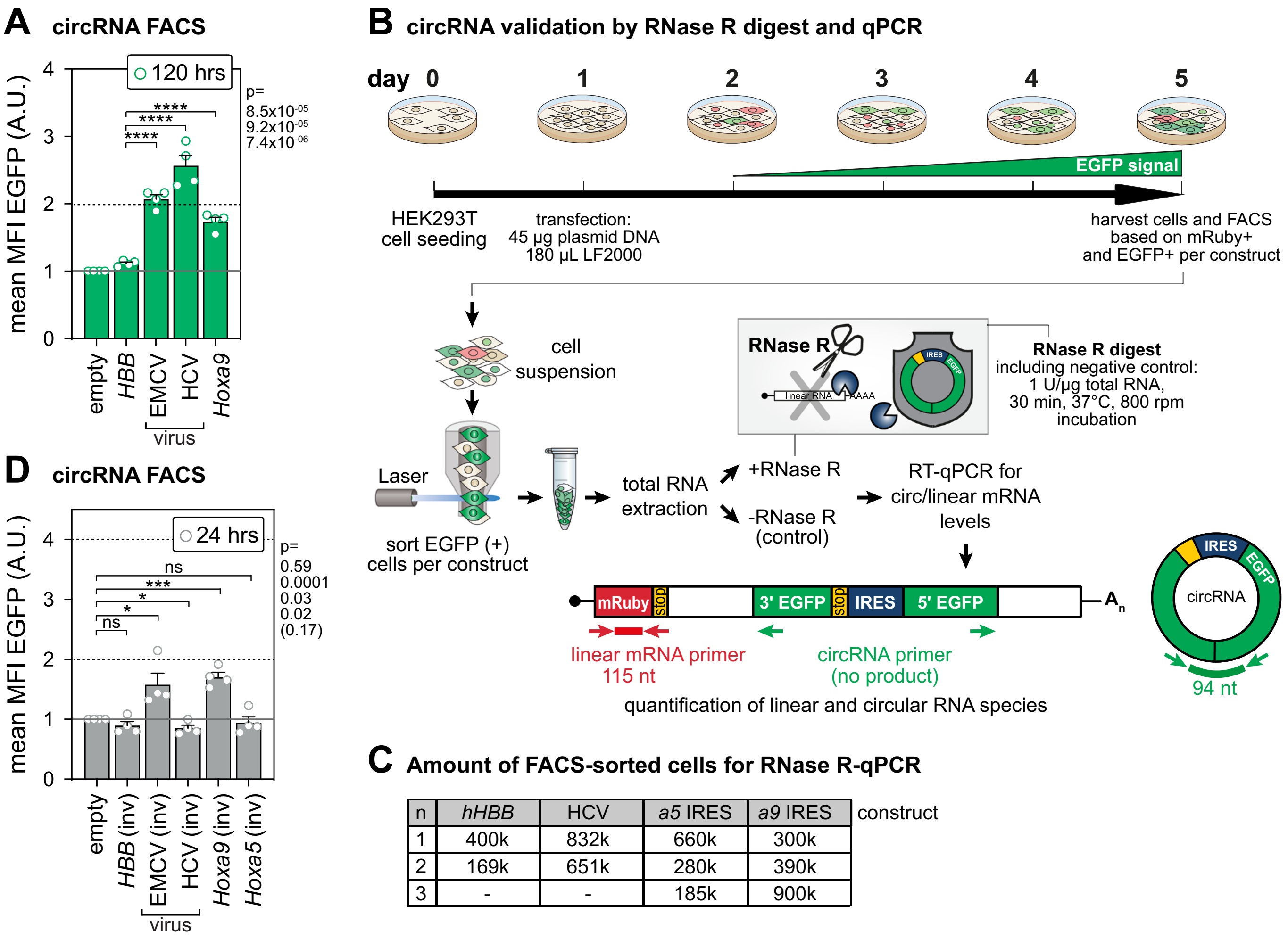

C Amount of FACS-sorted cells for RNase R-qPCR

n	*hHBB*	HCV	*a5* IRES	*a9* IRES
1	400k	832k	660k	300k
2	169k	651k	280k	390k
3	-	-	185k	900k

construct

Figure EV3. circRNA detection after plasmid transfection, RNase R cleanup and RT-qPCR.

(**A**) Calculated median fluorescence intensities (MFIs) of the mRuby+ subfractions are shown after normalization to the empty vector control in dependency of the tested insert sequences 120 h post transfection. Bar graphs are indicating mean values ± SEM, $n = 4$. See also Fig. 2B. (**B**) Experimental outline to proof the circRNA content of the HEK293T cells, generated by spliceosome-mediated backsplicing, after plasmid DNA transfection in order to validate the origin of the observed EGFP signal. Transfected cells were harvested 5 days post transfection and FACS-sorted according to their mRuby/EGFP signal. Afterwards, total RNA extraction was performed on the double positive cell fraction and subsequently digested with RNase R (1 U/µg RNA) for 30 min at 37 °C. The negative control was incubated with RNase R reaction buffer. qPCR was used for final circRNA quantification. EGFP primer will only lead to a product of 94 nt length after successful backsplicing. The 115 nt-long mRuby product was used for linear pre-mRNA quantification. The enrichment of circRNA over the RNase R digestion is shown in Fig. 2D. (**C**) Cell numbers of the EGFP+ cell fractions of the indicated samples shown for the $n = 2$–3 experiments used for RNase R-qPCR. (**D**) Calculated mean fluorescence intensities (MFIs) of the mRuby+ subfractions are shown after normalization to the empty vector control in dependency of the tested insert sequences 24 h post transfection. Bar graphs are indicating mean values ± SEM, $n = 4$. See also Fig. 2E. Source data are available online for this figure.

 © The Author(s)

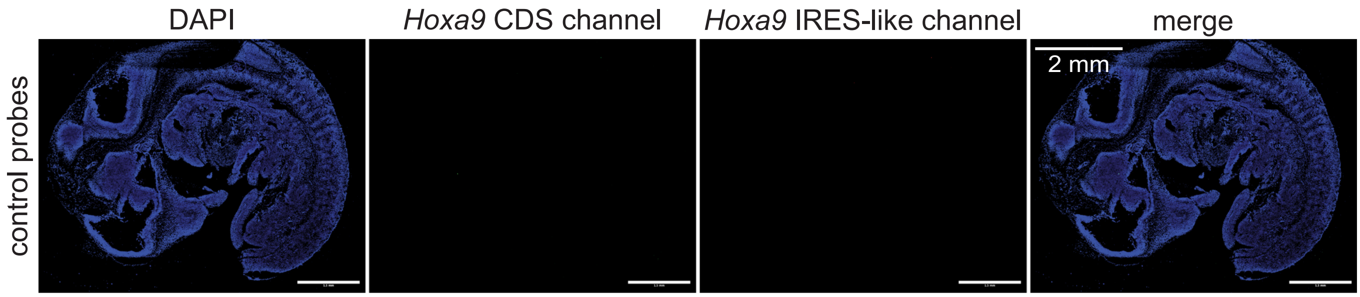

Figure EV4. Low background is observed for smFISH with control probes.

Representative images of the E11 mouse embryo sagittal sections stained with commercially available RNAscope Negative Control Probes targeting the *DapB* gene. Low background is observed in the channels used for visualizing *Hoxa9* CDS and IRES-like regions. This control probe staining was performed in parallel to the specific probe staining Fig. 3B in the same experiment but Fig. 3B and the panel shown here represent different embryo sections.

© The Author(s)

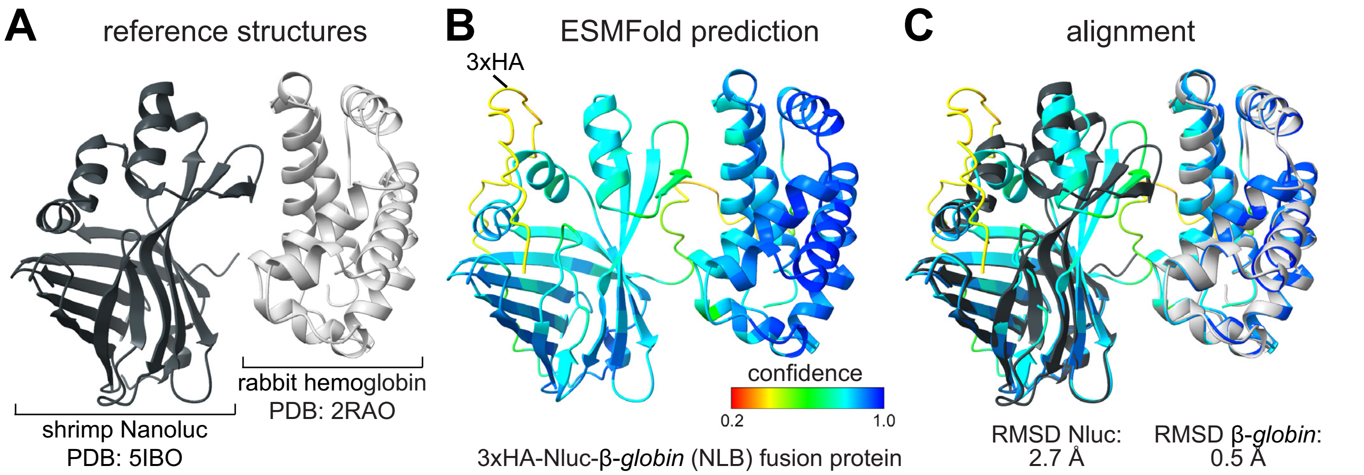

Figure EV5. EMSFold structure prediction of the NLB reporter fusion protein.

(**A**) For comparison, the crystal structure of shrimp Nanoluc (Nluc, PDB: 5IBO) and chain B of rabbit *hemoglobin* (PDB: 2RAO) are shown, in dark and light gray, respectively. Nluc and rabbit *hemoglobin* fold into a β-barrel and a globular fold, respectively, of similar size. (**B**) EMSFold, a large language model for protein structure prediction with evolutionary information (Lin et al, 2023), yields a high confidence prediction of the structure of the designed NLB reporter (average per-residue model confidence score plddt of 0.78). Structure prediction of the fusion protein NLB with the flexible 3xHA tag and interdomain linker reveals that both Nluc and β-*globin* can adopt their native folds, with a less compact thus less stable fold of the Nluc β-barrel, while the flexible linker and N-terminal 3xHA tag are unstructured. Predicted structure is colored by the model confidence. (**C**) Crystal structures aligned to the respective domains in the NLB reporter and the root-mean-square deviation (RMSD) for each of them are provided. The alignment of the predicted NLB fusion protein with the individual crystal structures reveals that in the fusion protein, the β-barrel of the Nluc is less compact in the fusion protein, suggesting that the fold is less stable within the proposed construct. Given that the luciferase activity of Nluc is tightly linked to substrate oxidation in its central cavity for luminescence (Tomabechi et al, 2016), this effect on the native Nluc folding state may explain the reduced activity of NLB.

 © The Author(s)
